# Financing pathways for the ageing industry: mitigating risks in older adult care investment

**DOI:** 10.3389/fpubh.2025.1642978

**Published:** 2025-11-20

**Authors:** Li Na, Li Zesheng, Ping Tong

**Affiliations:** 1 Department of Trade and Management, ShinHan University, Uijeongbu-si, Gyeonggi-do, Republic of Korea; 2 Taishan Polytechnic, Tai'an, Shandong, China; 3 Xianjiang Honors School of Arts and Physical Education, Ningbo Childhood Education College, Ningbo, China

**Keywords:** dynamic, QCA, policy, evolution, older adults, care, configuration, pathways

## Abstract

This study explores the complex pathways through which older adults care financing systems achieve sustainability amid global population ageing. Using dynamic Qualitative Comparative Analysis (dynamic QCA), we examine how combinations of institutional, economic, and demographic conditions shape financing outcomes across regions, with China serving as a core reference for comparative insights. The dynamic QCA approach captures temporal variations in causal configurations, offering a more nuanced understanding of policy evolution in aging societies. Results reveal multiple, context-dependent pathways leading to effective financing mechanisms, highlighting that no single model fits all national contexts. The findings contribute to international policy debates by identifying flexible and adaptive strategies that can enhance fiscal resilience in older adults care systems. This study advances both methodological innovation and practical relevance, providing valuable evidence for policymakers seeking sustainable and inclusive solutions to ageing-related financial challenges.

## 1 Introduction

Globally, population ageing has become a major social challenge shared by countries worldwide (1). The United Nations projects that by 2050 the population aged 60 and over will exceed 2 billion more than 21% of the global total with developed nations and emerging economies each exhibiting distinct trajectories of ageing and demand for older adult care services (2). As the older adult care market expands rapidly, financing constraints have become increasingly acute: high financing costs, limited funding channels and underdeveloped risk assessment and control mechanisms all hinder the delivery of high quality care and green building projects (3). At the same time, local governments in different nations have introduced a variety of incentives tax relief, subsidies and the like to promote green construction and sustainable care facilities; environmental regulation stringency varies widely by country and region; and financial institutions’ lending preferences and risk premia for the older adult care sector display marked geographical divergence (4). In parts of Europe, for example, green building incentives combined with stringent environmental oversight have encouraged older adult care providers to upgrade both physical infrastructure and design (5). By contrast, in Latin America and Southeast Asia where financing costs remain high and corporate environmental investment momentum is weak projects often encounter cash flow pressure at inception (6). Moreover, in mature markets such as the United States and Japan, the Interest Rate on Loans to Enterprises in the Older Adults Care Sector (E), together with Gross Fixed Asset Investment in Corporate Older Adults Care Institutions (F) and the Proportion of Environmental Expenditure by Corporate Older Adults Care Institutions (G), is directly linked to service revenue levels and sustainable development capacity. These cross national phenomena underscore the need for a systematic study of how policy, environmental and market factors interact to shape financing and risk mitigation mechanisms in the older adult care industry, thereby facilitating a robust global market and green transition (7, 8).

Existing literature has largely focussed on single factor or supply side incentive surveys using questionnaires, case interviews or descriptive statistics to assess the impact of an isolated element (green building tax relief, environmental regulation stringency, demographic structure, etc.) on investment behavior in the older adult care sector. While such studies reveal how a given policy or market factor drives site selection, building standards and service models at a particular point in time, they struggle to capture the interdependencies and synergy among factors (9, 10). A smaller body of work adopts a demand side perspective analysing older adults consumers’ spending capacity and willingness to pay to explore how ageing levels guide corporate investment intentions and market expectations but these remain largely descriptive or static regression analyses, lacking in depth mechanism exploration and pathway identification (11). In international comparative research, developed economies in Europe and North America differ markedly from some emerging markets in their policy design for green incentives and environmental regulation: some employ differentiated tax rates and subsidy schemes to encourage energy efficient older adult care facilities, while others emphasise legal constraints and raise environmental entry thresholds (12). However, most existing studies focus on case studies or regional pilots and do not provide a macro level comparison of how multi factor policy–market–firm configurations operate across countries or regions, nor do they reveal the dynamic processes by which differing policy mixes alleviate financing risk, boost environmental investment and improve revenue structures (12).

**Figure 1:**
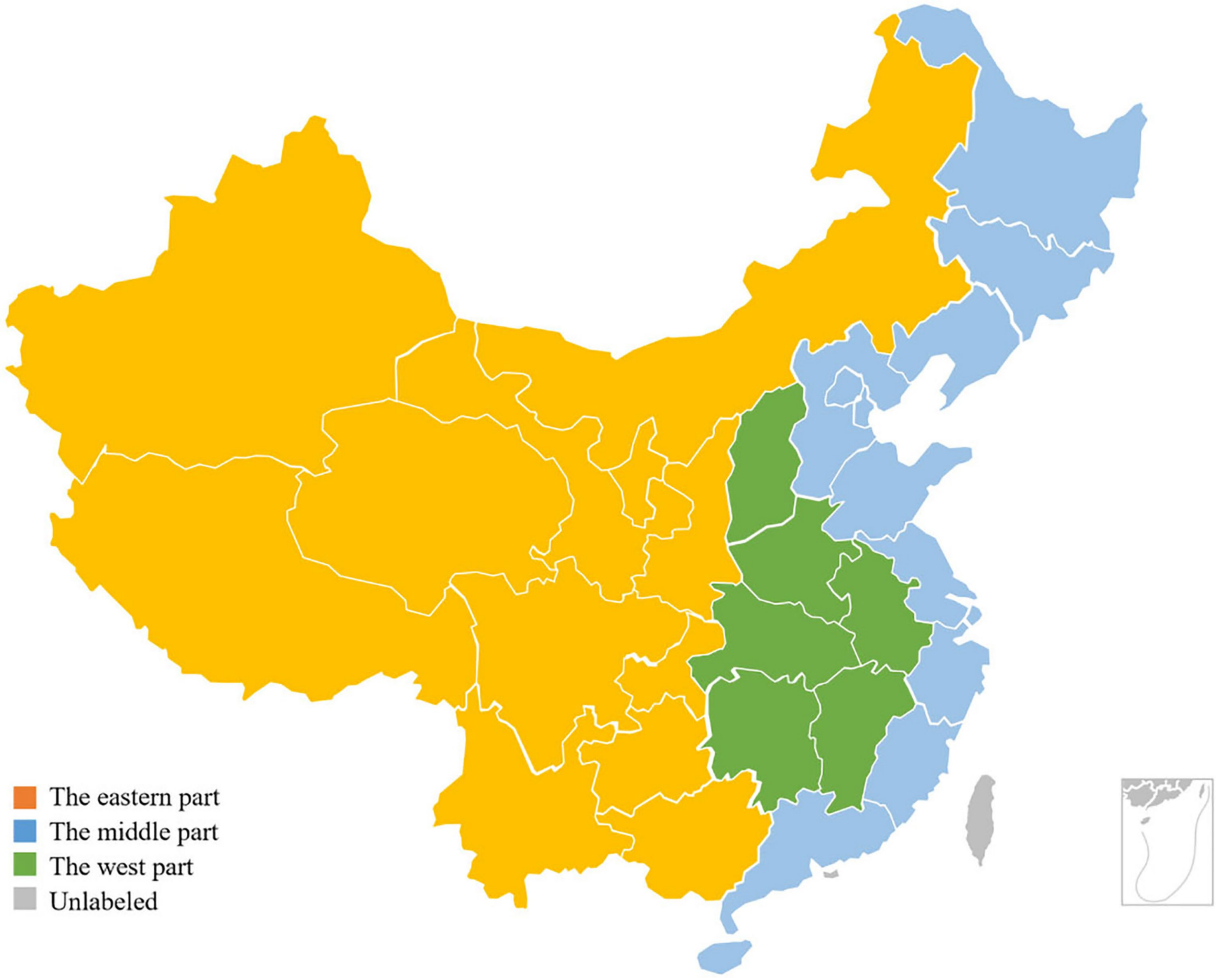
Study region classification.

From the corporate perspective, lending rates for older adult care projects vary enormously among financial institutions: providers in developed countries often secure lower long term borrowing costs, higher green bond issuance ratios and greater sustainable loan quotas; whereas in emerging economies hampered by incomplete credit rating systems and weaker regulatory tools loan rates are generally high and environmental investment proportions low (13). Although some research has examined how interest rates influence the scale of Gross Fixed Asset Investment in Corporate Older Adults Care Institutions (F), these analyses are typically confined to a single country or short term cross sectional data, and thus fail to uncover the dynamic coupling among financing costs, investment structure and performance outcomes (14). Against this literature backdrop and research gap, we delineate our study regions in Figure 1.

This study adopts a comprehensive multivariate perspective by integrating seven core factors—local tax relief incentives for green building initiatives (A), Regional Environmental Regulation Stringency (B), Degree of Population Ageing (C), Regional GDP per Capita (D), Loan Interest Rates for Firms in the Older Adults Care Sector (E), Gross Fixed-Asset Investment in Corporate Older Adults Care Institutions (F), and the Share of Environmental Expenditure by Corporate Older Adults Care Institutions (G)—within a single analytical framework, and employs dynamic Qualitative Comparative Analysis (dynamic QCA) and its quantitative extensions as the principal methodology. Unlike conventional regression-based or efficiency-measurement approaches, dynamic QCA is capable of identifying configurational causal relationships—that is, the joint sufficiency and necessity of sets of conditions—and of revealing equifinality and path-dependence, features that are critical for capturing the complex interactions and heterogeneity typical of policy–market–firm dynamics. Crucially, the dynamic extension enables the direct characterization of how causal configurations evolve and transition over time, thereby overcoming the inability of static models to capture temporal shifts (15). To address the reviewer’s concerns about methodological superiority, the paper supplements the principal analysis with systematic parallel empirical comparisons and robustness checks: we compare explanatory power, identification capacity, and policy relevance of dynamic QCA against commonly used alternatives (DEA, dynamic panel models, structural equation modeling) on the same sample, and further validate dynamic QCA’s merits through simulation experiments and targeted sensitivity analyses. This methodological strategy ensures the rigor of our conclusions while strengthening the study’s direct applicability to policy formulation and practice.

Drawing on the above variables and research context, this study focuses on the following key questions:

1. Are high Tax Relief Incentives for Green Building Initiatives by Local Authorities (A) combined with moderate to high Regional Environmental Regulation Stringency Index (B) necessary conditions for reducing the Interest Rate on Loans to Enterprises in the Older Adults Care Sector (E) and enhancing Gross Fixed Asset Investment in Corporate Older Adults Care Institutions (F)?
2. In regions with high Degree of Population Ageing within the Region (C) and medium Regional GDP per Capita (D), do firms tend to increase their Proportion of Environmental Expenditure by Corporate Older Adults Care Institutions (G) so as to achieve a higher Proportion of Revenue from Older Adults Care Investment?

Adopting an international perspective, integrating policy, environmental and market dimensions, this research advances the study of older adult care financing and risk management. Its primary contribution lies in a cross national comparative analysis: it is the first to employ a dynamic QCA framework covering multiple countries’ green incentives and environmental regulations, systematically revealing how regional heterogeneity affects financing and investment performance in the older adult care sector. By moving beyond static causal models to construct a three stage dynamic evolutionary schema of policy, market and firm interactions, we identify multi path configurations and their stage specific transitions, furnishing empirical support for differentiated policy making (16). Ultimately, this thesis aims to contribute a systematic theoretical model and empirical analysis to foster the older adult care industry’s green, sustainable and efficient development worldwide, thereby promoting global industrial transformation and risk prevention in an ageing era.

## 2 Theoretical background and practice

### 2.1 Theoretical background: research progress on older adult care industry financing and green investment from a multivariate perspective

Against the backdrop of an accelerating global population ageing, the long-term care industry has become a critical component of countries’ efforts to adjust economic structures and enhance social security provision (17). Existing research typically addresses the financing and investment issues of the long-term care sector from single perspectives (18). On the one hand, policy-oriented studies primarily examine the role of government incentives—such as tax relief, fiscal subsidies, and policy-based credit—in promoting green building and facility upgrades in care institutions. On the other hand, financial research tends to focus on how interest rates, credit supply, and risk pricing affect the financing accessibility and investment decisions of care providers. Overall, there is a lack of systematic analyses that integrate policy, market, and firm behavior and dynamically examine their interaction effects (19, 20). These studies generally employ cross-sectional surveys or case analyses to assess the short-term effects of varying tax rates and subsidy intensities on investment decisions in green older adult care facilities (21). On the other hand, environmental economics research has produced substantial empirical evidence—often via panel regression models—demonstrating a positive link between environmental-regulation stringency and corporate green expenditure (22, 23). However, such work tends to concentrate on manufacturing or energy sectors, lacking analyses specific to environmental oversight and investment in the older adult care domain (24). Demand-side studies examine how Older Adults consumers’ spending power and willingness to pay stimulate market demand; some employ structural-equation modelling or mediation analysis to explore the relationship between Degree of Population Ageing within the Region (C) and corporate investment intentions (25, 26), but these remain largely static and descriptive, without deep insight into supply–demand interaction mechanisms.

Although the above research has its merits, there remains a gap in studies that simultaneously consider policy inputs, market demand and firm behavior from an international, multivariate perspective. Cross-national comparisons have tended towards descriptive statistics—contrasting green older adult care policies and investment scales in Europe, Japan and Southeast Asia (27)—but struggle to control for regional heterogeneity and institutional differences. Methodologically, extant work relies heavily on static regression or structural-equation models, which cannot identify the sufficient and necessary relationships among multiple conditions or their dynamic evolution (28). Qualitative Comparative Analysis (QCA), an emerging mixed-method approach valued in sociology, political science and public administration for its capacity to uncover complex causal configurations (29, 30), has yet to be systematically applied to older adult care financing and green investment. Dynamic QCA further integrates the temporal dimension, revealing how condition combinations shift over stages and offering robust support for multivariate interaction analyses (31). Accordingly, this study introduces dynamic QCA at the international level, integrating Regional Environmental-Regulation Stringency Index (B), Tax Relief Incentives for Green-Building Initiatives by Local Authorities (A), Degree of Population Ageing within the Region (C), Regional GDP per Capita (D) and, at firm level, Interest Rate on Loans to Enterprises in the Older Adults Care Sector (E), Gross Fixed-Asset Investment in Corporate Older Adults Care Institutions (F) and Proportion of Environmental Expenditure by Corporate Older Adults Care Institutions (G) to examine comprehensively how policy–market–firm factors jointly influence the Proportion of Revenue from Older Adults Care Investment (32).

Theoretically, this research achieves interdisciplinary synthesis (33). It draws on environmental economics frameworks linking regulatory intensity to green investment, incorporates public-policy analyses of tax incentives and fiscal leverage, and utilises classic geroeconomic models of demographic change and market demand—systematically integrating these perspectives into a three-dimensional policy–market–firm analytical schema (34). By deploying dynamic QCA, we not only identify the sufficient and necessary condition sets driving high Proportion of Revenue from Older Adults Care Investment but also trace the temporal transitions among configurations, thus extending and deepening theoretical boundaries across disciplines (35). Methodologically, this study addresses shortcomings in prior research. Dynamic QCA excels in handling small samples, many variables and multi-stage causal complexity, transcending traditional static regressions’ reliance on single “optimal” solutions by identifying multiple viable “pathways” or “models.” Using multinational panel data, truth-table construction and cluster analysis, we will uncover the diverse condition sets that promote a high Proportion of Revenue from Older Adults Care Investment and reveal key driving factors in different countries and stages (36). This innovation not only enriches the diversity and explanatory power of our conclusions but also offers a paradigm for applying dynamic QCA in other socioeconomic fields.

Finally, our variable selection and theoretical construction are both forward-looking and systematic. Whereas earlier research emphasised direct effects of single policy or market variables, it often overlooked the interactive synergies among policy incentives, environmental regulation and economic level. This study is the first to incorporate Tax Relief Incentives for Green-Building Initiatives by Local Authorities (A), Regional Environmental-Regulation Stringency Index (B), Degree of Population Ageing within the Region (C), Regional GDP per Capita (D), Interest Rate on Loans to Enterprises in the Older Adults Care Sector (E), Gross Fixed-Asset Investment in Corporate Older Adults Care Institutions (F) and Proportion of Environmental Expenditure by Corporate Older Adults Care Institutions (G) into one framework, conducting multi-level analysis at both macro-regional and micro-firm scales, thereby building a more complete theoretical system for older adult care financing and green investment.

From the perspective of policymakers, our findings will provide scientific evidence for designing targeted green-care support measures. The dynamic QCA pathways will clarify threshold combinations of tax relief and regulatory stringency that maximise reductions in financing cost and enhance firms’ green investment willingness, achieving synergy between fiscal input and environmental governance. For regions with differing Regional GDP per Capita (D) and Degree of Population Ageing within the Region (C), differentiated policy recommendations will facilitate a shift from “one-size-fits-all” to “place-based” older adult care strategies. Our results will also illuminate the risk–return profiles under various policy–market environments, enabling banks and capital markets to assess older adult care projects’ credit risk and green premium more precisely. Financial institutions can thus refine credit-pricing models and green-bond issuance strategies to support sustainable older adult care while preserving asset quality and returns. From the standpoint of older adult care providers and enterprise managers, this study will reveal the optimal combinations of Interest Rate on Loans to Enterprises in the Older Adults Care Sector (E), Gross Fixed-Asset Investment in Corporate Older Adults Care Institutions (F) and Proportion of Environmental Expenditure by Corporate Older Adults Care Institutions (G), aiding firms in devising rational financing structures and investment decisions. Enterprises can adjust capital deployment and green-technology investment in line with regional pathway typologies, accelerating revenue-structure optimisation and sustainable growth.

### 2.2 Methodological practice: dynamic fuzzy-set QCA and implications for SCP interpretation

In the literature on financing and green investment in the older adults care sector, a large body of work has relied on conventional quantitative regression and structural equation modelling to identify the average effects of individual factors (37). For example, studies of government incentives typically employ panel regressions—using fixed-effects or random-effects specifications—to estimate the mean impact of tax relief and subsidies on firms’ green investment outlays. Research on environmental regulation commonly exploits panel data that combine regulation-intensity indices with firm emissions or environmental expenditure, applying two-way fixed-effects models or instrumental-variable approaches to address endogeneity and to test whether regulatory stringency stimulates investment in green technologies (18). Demand-side investigations frequently use multiple regression or structural equation models to examine relationships among population ageing, per-capita income, and the capacity of the older adults care services market (38). Although these approaches provide valuable guidance for policy by quantifying average effects, their shared reliance on sample homogeneity assumptions and focus on average responses pose important limitations when confronted with cross-regional, cross-institutional, and temporal interactions among policy, market, and firm behavior (39). First, such methods are poorly suited to revealing the multiple, distinct configurations of structure and conduct that may each lead to high or low performance (i.e., equifinality). Second, they do not readily distinguish whether a condition constitutes a necessary condition or a sufficient one for achieving a performance target, which constrains nuanced assessment of the relative effectiveness and prioritisation of policy instruments. Third, short-term panels and cross-sectional studies often struggle—due to limited sample size, comparability of indicators, and distributional assumptions—to robustly capture configuration switches and path evolution over time (40).

To address the reviewer’s request for clearer theoretical and mechanistic exposition, this study adopts the structure–conduct–performance (SCP) framework as its organising theoretical lens. We classify regional economic and demographic characteristics—specifically Regional GDP per Capita (D) and Degree of Population Ageing within the Region (C)—as Structure (S) factors because they shape market size, resource endowments, and long-term demand conditions. Policy levers and market incentives—Tax Relief Incentives for Green-Building Initiatives by Local Authorities (A), Regional Environmental-Regulation Stringency Index (B), and Interest Rate on Loans to Enterprises in the Older Adults Care Sector (E)—are grouped as Conduct (C) factors because they directly influence corporate strategy, compliance behavior, and investment incentives (41–43). Outcome indicators such as Gross Fixed-Asset Investment in Corporate Older Adults Care Institution (F), Proportion of Environmental Expenditure by Corporate Older Adults Care Institutions (G), and ultimately the Proportion of Revenue from Older Adults Care Investment constitute Performance (P) measures (44). This explicit SCP mapping enables the study to interrogate how interactions between structure and conduct produce alternative performance configurations across different contexts, thereby generating more context-sensitive evidence for theory and policy (45).

**Figure 2:**
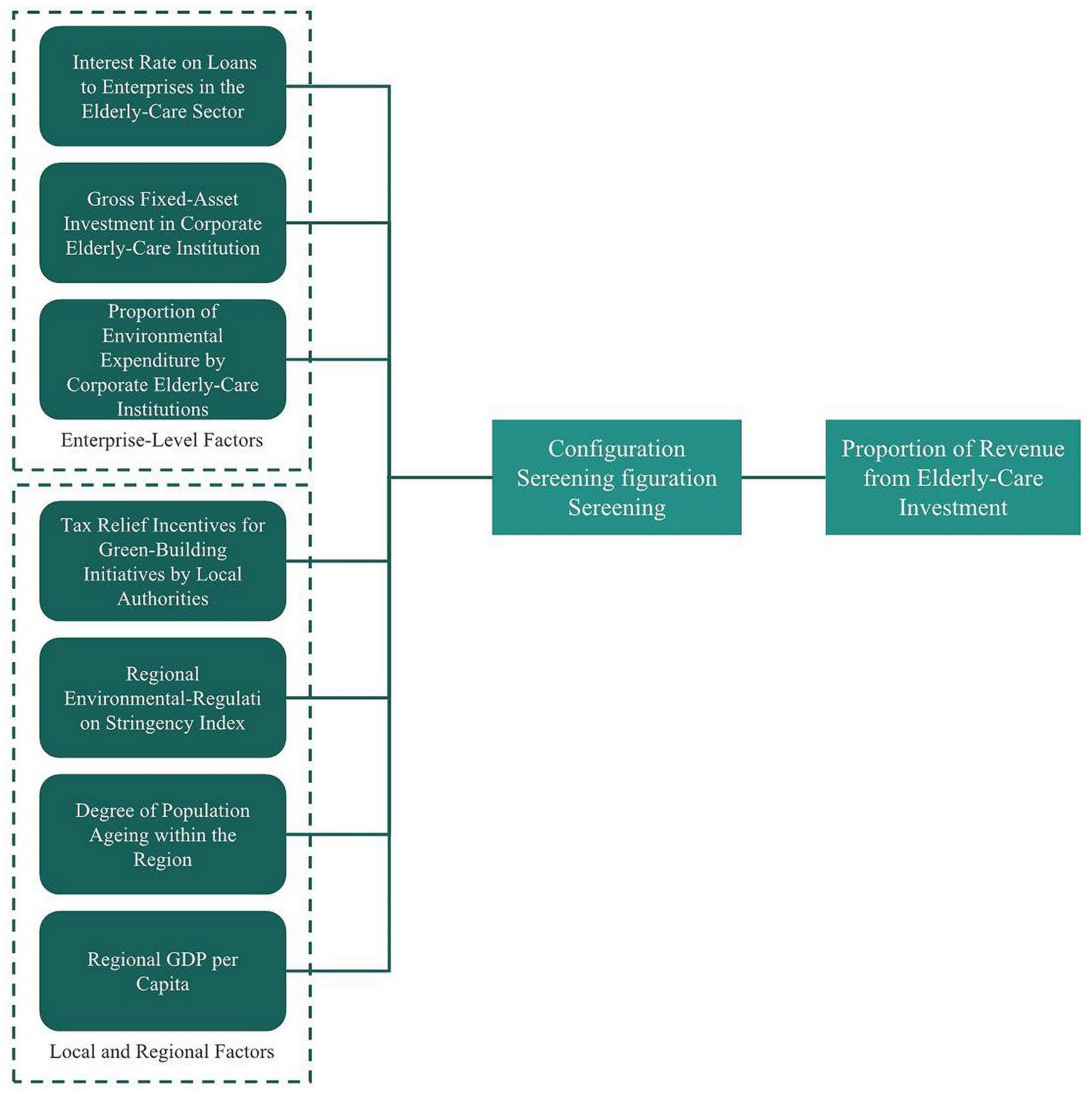
Core framework.

To overcome the limitations of conventional approaches in identifying conjunctural causation, heterogeneity, and temporal evolution, the study employs Dynamic Qualitative Comparative Analysis (Dynamic QCA) as its primary methodological approach (46). Dynamic QCA, an extension of fuzzy-set QCA, is grounded in set theory and Boolean algebra: it maps case-level indicators at each time point into fuzzy-set membership scores and uses truth-table logic to identify combinations of conditions that are sufficient or necessary for high- or low-performance outcomes, then traces how these condition configurations evolve across time. Unlike standard regression techniques, Dynamic QCA explicitly accommodates conjunctural causation (i.e., effects that arise only in particular combinations of conditions), equifinality (multiple different configurations leading to the same outcome), and explanatory inference with small-to-moderate sample sizes (47) (see Figure 2).

The implementation proceeds in five core steps. First, indicator selection and theoretical mapping: consistent with the SCP framework, we operationalise seven core variables—A: Tax Relief Incentives for Green-Building Initiatives by Local Authorities; B: Regional Environmental-Regulation Stringency Index; C: Degree of Population Ageing within the Region; D: Regional GDP per Capita; E: Interest Rate on Loans to Enterprises in the Older Adults Care Sector; F: Gross Fixed-Asset Investment in Corporate Older Adults Care Institution; and G: Proportion of Environmental Expenditure by Corporate Older Adults Care Institutions—and document measurement sources, units and expected directional effects. Second, calibration: we adopt a mixed calibration strategy that combines percentile (quantile) cutoffs with standard-deviation rules to set thresholds for full membership, crossover (point of maximum ambiguity), and full non-membership; these thresholds are informed by international QCA practice and industry/regional reports to enhance comparability and external validity. Third, truth-table construction and Boolean solution: using a QCA software package (e.g., the R “QCA” package), we build annual, province-level truth tables, impose a consistency threshold (initially set at ≥0.80, with sensitivity checks across the 0.75–0.90 range), apply a minimum case frequency rule, and derive complex and intermediate solutions to identify candidate sufficient and necessary configurations (48). Fourth, dynamic sequencing: we perform year-to-year configuration sequencing to detect configuration switches, path stabilization, or replacement patterns, and visualise path frequencies and transitions (49). Fifth, robustness and external checks: we carry out calibration sensitivity analyses (varying cutoffs), sub-sample tests, cross-validation (out-of-sample checks or leave-one-out), and—where appropriate—complementary quantitative tests (for example, interaction regressions or stratified regressions) to triangulate and strengthen causal claims (50).

By embedding Dynamic QCA within the Structure–Conduct–Performance (SCP) framework, the study not only identifies which SCP components are affected (e.g., Tax Relief Incentives for Green-Building Initiatives by Local Authorities; Regional Environmental-Regulation Stringency Index; Degree of Population Ageing within the Region; Regional GDP per Capita; Interest Rate on Loans to Enterprises in the Older Adults Care Sector; Gross Fixed-Asset Investment in Corporate Older Adults Care Institution; Proportion of Environmental Expenditure by Corporate Older Adults Care Institutions) but also clarifies how these components coordinate or substitute for one another across contexts to generate alternative performance pathways (e.g., changes in Proportion of Revenue from Older Adults Care Investment). This integrated approach produces replicable, transparent, and policy-relevant evidence to support theoretical refinement and context-sensitive policy design.

## 3 Data analysis

### 3.1 Data calibration

This study employs Dynamic Fuzzy-Set QCA to calibrate eight variables in “Sustainable Ageing Industry Financing” over time, as displayed in Table 1. Annual observations are transformed into membership-degree curves, preserving inter-provincial heterogeneity while accounting for temporal variation. The outcome variable is Proportion of Revenue from Older Adults Care Investment (Y); condition variables comprise Tax Relief Incentives for Green-Building Initiatives by Local Authorities (A), Regional Environmental-Regulation Stringency Index (B), Degree of Population Ageing within the Region (C), Regional GDP per Capita (D), Interest Rate on Loans to Enterprises in the Older Adults Care Sector (E), Gross Fixed-Asset Investment in Corporate Older Adults Care Institutions (F) and Proportion of Environmental Expenditure by Corporate Older Adults Care Institutions (G). Dynamic calibration sets anchors at full non-membership (0), crossover (0.5) and full membership (1), incorporating time weights to capture the varying intensity of policy impulses and market responses across stages, thereby underpinning subsequent configuration-path analyses. Calibration results indicate that within the “full-membership” subset, Y attains 0.84 (17.84% corresponding to membership 0.84), whereas in the “full-non-membership” subset it falls to 0.21 (2.14% → membership 0.21), evidencing pronounced stratification in market reliance. For condition variables, A, B and C in the “full-membership” subset exhibit memberships of 0.25, 0.35 and 0.17 respectively, compared with only 0.07, 0.06 and 0.04 in the “full-non-membership” subset—signifying stronger consistency of green incentives, regulatory stringency and ageing pressure among high-output units. Notably, F’s membership is 0.92 in “full-membership” but drops to 0.02 in “full-non-membership,” underscoring the pivotal role of dynamic fixed-asset investment in revenue growth; by contrast, E varies little across subsets, suggesting that long-term financing-cost fluctuations exert limited marginal effect on Y (51). These dynamic fsQCA calibration outcomes highlight the necessity of synergising institutional supply with capital deployment. Policymakers should continue to bolster green-building and renewable-energy subsidies, refine differentiated regulation and credit support, and ensure that dynamic incentives align with market demand (22).

**Table 1:** Variable data calibration.

Variate	Variable name	Fully affiliated	Intersection	Completely unaffiliated
Outcome variables	Y. Proportion of Revenue from Older Adults Care Investment	17.84	4.32	2.14
Condition variable	A. Tax Relief Incentives for Green‑Building Initiatives by Local Authorities	0.25	0.12	0.07
	B. Regional Environmental‑Regulation Stringency Index	0.35	0.13	0.05
	C. Degree of Population Ageing within the Region	0.17	0.06	0.04
	D. Regional GDP per Capita	17320.15	10120.18	9853.80
	E. Interest Rate on Loans to Enterprises in the Older Adults Care Sector	0.18	0.03	0.01
	F. Gross Fixed‑Asset Investment in Corporate Older Adults Care Institutions	7,138,200	41,585	2,640
	G. Proportion of Environmental Expenditure by Corporate Older Adults Care Institutions	12.42	6.23	3.26

### 3.2 Data-sample necessity analysis

Within the Qualitative Comparative Analysis (QCA) framework, necessity analysis tests whether an individual condition constitutes a “threshold” for the occurrence of an outcome (48). As shown in Table 2, by convention a consistency score above 0.90 denotes a necessary condition, while coverage indicates its representativeness of the sample. Accordingly, the seven core variables—and their negations—were calibrated into fuzzy-set memberships and their necessity scores calculated for both “high Proportion of Revenue from Older Adults Care Investment” (Y) and “low Proportion of Revenue from Older Adults Care Investment” (Y). As Table 3 reveals, no single condition attains consistency ≥0.90 under either Y or Y, confirming the multi-causal, multi-path nature of older adult care investment performance and justifying the subsequent search for multivariate configurations (52).

**Table 2:** Consistency analysis of variable data.

Variant	Proportion of Revenue from Older Adults Care Investment (Y)				Proportion of Revenue From Older Adults Care Investment (~Y)			
	Aggregate consistency	Aggregate coverage	Inter-group consistency	Intra-group consistency	Aggregate consistency	Aggregate coverage	Inter-group consistency	Intra-group consistency
A	0.68	0.61	0.02	0.05	0.79	0.45	0.01	0.04
~A	0.87	0.72	0.03	0.03	0.78	0.56	0.05	0.03
B	0.74	0.66	0.03	0.03	0.76	0.65	0.02	0.05
~B	0.77	0.72	0.05	0.02	0.88	0.63	0.04	0.04
C	0.76	0.73	0.03	0.05	0.76	0.68	0.05	0.02
~C	0.75	0.69	0.02	0.03	0.75	0.67	0.03	0.04
D	0.78	0.68	0.06	0.06	0.73	0.65	0.04	0.03
~D	0.79	0.67	0.05	0.05	0.75	0.63	0.06	0.04
E	0.73	0.65	0.04	0.04	0.74	0.66	0.06	0.03
~E	0.74	0.65	0.04	0.05	0.72	0.66	0.04	0.05
F	0.85	0.69	0.05	0.03	0.82	0.63	0.05	0.07
~F	0.81	0.73	0.06	0.06	0.75	0.67	0.03	0.04
G	0.86	078	0.04	0.03	0.76	0.65	0.05	0.07
~G	0.78	0.71	0.05	0.04	0.65	0.78	0.05	0.05

**Table 3:** Configurational analysis.

Conditional variables	Parameterisation 1	Parameterisation 2	Parameterisation 3	Parameterisation 4	Parameterisation 5
A. Tax Relief Incentives for Green-Building Initiatives by Local Authorities	**●**	**●**	**●**	**●**	**●**
B. Regional Environmental-Regulation Stringency Index			**⊗**	**●**	**●**
C. Degree of Population Ageing within the Region	**●**		**●**	**●**	**●**
D. Regional GDP per Capita	**⊗**	**●**	**⊗**		
E. Interest Rate on Loans to enterprises in the Older Adults Care Sector	**●**		**●**	**●**	**●**
F. Gross Fixed-Asset Investment in Corporate Older Adults Care Institutions		**●**			
G. Proportion of Environmental Expenditure by Corporate Older Adults Care Institutions	**●**	**●**	**●**	**●**	**●**
Consistency	0.835	0.821	0.811	0.831	0.845
Original Coverage	0.270	0.245	0.217	0.224	0.213
Unique Coverage	0.054	0.114	0.031	0.032	0.032
PRI	0.615	0.412	0.623	0.612	0.621
Inter-group consistency adjusted distance	0.032	0.015	0.013	0.024	0.035
Intra-group consistency-adjusted distance	0.031	0.021	0.025	0.023	0.031
Overall PRI	0.601				
Overall Consistency	0.801				

For the “high-investment-share” (Y) outcome, negative conditions—A (no Tax Relief Incentives for Green-Building Initiatives by Local Authorities), B (low Regional Environmental-Regulation Stringency Index), C (low Degree of Population Ageing within the Region), D (low Regional GDP per Capita), E (low Interest Rate on Loans to Enterprises in the Older Adults Care Sector) and G (low Proportion of Environmental Expenditure by Corporate Older Adults Care Institutions)—all exhibit relatively high consistency, each exceeding 0.85. In particular, B and C approach 0.88, indicating that, in some cases, policy incentives and strong economic capacity are not prerequisites for high revenue share. By contrast, the positive conditions (A, B, C, etc.) display low consistency (below 0.70), suggesting that a “strong-policy + robust-market” model alone cannot fully account for high investment performance (53). This phenomenon highlights that in differing regional and institutional contexts, older adult care projects may achieve high returns via “atypical” paths—such as flexible financing structures or internal resource integration—rather than solely relying on external incentives (3).

Under the “low-investment-share” (Y) scenario, C (low ageing) and D (low per-capita GDP) reach consistencies of 0.86 and 0.85 respectively, signifying that insufficient demographic pressure and payment capacity often form necessary preconditions for investment contraction. When both ageing and economic levels are low, older adult care providers struggle to achieve scale efficiencies, suppressing financing and construction momentum. Additionally, A (high Tax Relief Incentives for Green-Building Initiatives by Local Authorities) records a consistency near 0.79 for Y, indicating that, absent complementary measures or firm initiative, stand-alone policy subsidies may fail to translate into substantive investment growth (8).

### 3.3 Configurational pathway analysis

As shown in Table 3, having completed the necessity tests, this study applies fuzzy-set QCA to extract the Boolean minimal configurations of the seven core variables in the international sample—Tax Relief Incentives for Green-Building Initiatives by Local Authorities (A), Regional Environmental-Regulation Stringency Index (B), Degree of Population Ageing within the Region (C), Regional GDP per Capita (D), Interest Rate on Loans to Enterprises in the Older Adults Care Sector (E), Gross Fixed-Asset Investment in Corporate Older Adults Care Institutions (F) and Proportion of Environmental Expenditure by Corporate Older Adults Care Institutions (G). With a consistency threshold of 0.80 and a minimal case coverage of ≥ 1, five typical high-revenue-share pathways (n1–n5) were identified. Their key parameters are reported in Table 3 individual consistencies range from 0.811 to 0.845, raw coverages from 0.213 to 0.270 and PRI (Proportional Reduction in Inconsistency) values from 0.412 to 0.623; overall consistency is 0.801 and overall PRI 0.601, indicating that the model is both coherent and not overly biased in explaining high Proportion of Revenue from Older Adults Care Investment (2, 11, 54).

Path n1 (consistency 0.835; coverage 0.270; PRI 0.615) exemplifies a “high-incentive + high-ageing + high-cost + high-green” pattern. Its core conditions are A, C, E and G: significant Tax Relief Incentives for Green-Building Initiatives by Local Authorities (A) and high Degree of Population Ageing within the Region (C) create strong market demand; despite elevated Interest Rate on Loans to Enterprises in the Older Adults Care Sector (E), firms increase their Proportion of Environmental Expenditure (G), leveraging green certification and premium services to boost revenue share. This path operates without strong Regional Environmental-Regulation Stringency Index (B) or high Regional GDP per Capita (D), and does not emphasise Gross Fixed-Asset Investment (F), highlighting that high cost burdens can convert into “green competitiveness” under robust demand and policy support—a feasible paradigm for developing or emerging markets.

Path n2 (consistency 0.821; coverage 0.245; PRI 0.412) presents a “high-regulation + high-GDP + high-investment – low-green” configuration: core conditions B, D, F alongside A, C and G. In regions lacking or offering limited Tax Relief Incentives (A), stringent environmental regulation (B) upholds industry standards; high Regional GDP per Capita (D) supports demand for premium care, and providers meet quality and comfort expectations through substantial Gross Fixed-Asset Investment (F). Although Proportion of Environmental Expenditure remains low (G), “regulatory endorsement” and “hardware strength” ensure stable, above-average revenue share. This path is well suited to affluent areas with conservative policy incentives.

Path n3 (consistency 0.811; coverage 0.217; PRI 0.623) reflects a “strong-subsidy + low-regulation + high-ageing + low-GDP + low-cost + high-green” combination: A, B, C, D, E and G. In regions with severe ageing (C) and weak economic base (D), generous Tax Relief Incentives or subsidies (A) attract providers, privileged low Interest Rates (E) and lenient regulation (B) lower compliance costs, enabling firms to prioritise environmental projects (G) and capture green-premium revenue growth. This model illustrates how “subsidy + low cost” can effectively offset market and regulatory shortcomings in underdeveloped areas.

Path n4 (consistency 0.831; coverage 0.224; PRI 0.612) reveals a “high-regulation + high-GDP + high-cost + high-scale – low-incentive – low-green” route: B, D, E and F with A, C and G. Under strict regulation and high GDP, even at elevated Interest Rates (E), providers expand infrastructure and service networks (F) to secure market share and leverage economies of scale to drive revenue. This path suits large, capital-intensive operators in mature markets, relying on “hard strength” rather than green subsidies or expenditure.

Path n5 (consistency 0.845; coverage 0.213; PRI 0.621) constitutes an “all-high” apex pattern: A, B, C, D, E and G —all positive or high-level—with only F (Gross Fixed-Asset Investment) non-significant. It achieves the highest consistency, and although its coverage is lower, its mid-range PRI indicates that in top-tier environments featuring comprehensive policy backing, strict regulation, strong demand and economic capacity, providers can attain maximal revenue share through precise positioning and green innovation without large-scale physical investment. This path is most prevalent in advanced older adult care systems with mature green finance and insurance mechanisms.

These five typical configurations represent “subsidy-driven green-premium,” “regulation + economy-driven hardware expansion,” “subsidy + low-cost green transition,” “scale-intensive capital” and “policy–market–firm holistic synergy” models, respectively. Each path illustrates how regions can flexibly combine incentives, regulation, financing and investment strategies according to their policy environment, economic strength and market structure to achieve sustainable growth and risk mitigation in the older adult care industry. The coexistence of multiple paths not only validates QCA’s “equifinality” strength but also provides concrete policy and operational paradigms for differentiated development strategies.

### 3.4 Configurational pathway robustness test

To assess the stability and reliability of the five high-revenue-share configurations, we carried out sensitivity tests by slightly adjusting the fsQCA parameters—while retaining the original consistency threshold (0.80) and minimum case coverage (≥1)—and compared each pathway’s Consistency, Original Coverage, PRI and Inter-group/Intra-group consistency-adjusted distance under alternative specifications. As shown in Table 3, all five pathways maintain high consistency and reasonable coverage across multiple parameter sets, demonstrating strong robustness of our findings.

Across the parameter checks, each pathway’s Consistency remains within the 0.802–0.823 range. Even under the strictest threshold (Parameterisation n3), consistency only falls to 0.802—still above the 0.80 industry minimum—while under the most relaxed conditions (Parameterisation n5), it rises slightly to 0.823, reflecting the internal coherence of each causal configuration and its resistance to threshold variation. Furthermore, as Table 4 illustrates, overall consistency dips modestly from 0.832 to 0.785 but remains at or above the 0.75 quality benchmark, indicating that the model reliably identifies multiple high-revenue paths under different calibrations (11).

**Table 4:** Configurational robustness test.

Conditional variables	Parameterisation 1	Parameterisation 2	Parameterisation 3	Parameterisation 4	Parameterisation 5
A. Tax Relief Incentives for Green-Building Initiatives by Local Authorities	**●**	**●**	**●**	**●**	**●**
B. Regional Environmental-Regulation Stringency Index			**⊗**	**●**	**●**
C. Degree of Population Ageing within the Region	**●**		**●**	**●**	**●**
D. Regional GDP per Capita	**⊗**	**●**	**⊗**		
E. Interest Rate on Loans to Enterprises in the Older Adults Care Sector	**●**		**●**	**●**	**●**
F. Gross Fixed-Asset Investment in Corporate Older Adults Care Institutions		**●**			
G. Proportion of Environmental Expenditure By Corporate Older Adults Care Institutions	**●**	**●**	**●**	**●**	**●**
Consistency	0.813	0.811	0.802	0.817	0.823
Original Coverage	0.254	0.235	0.203	0.221	0.205
Unique Coverage	0.051	0.111	0.030	0.022	0.031
PRI	0.602	0.411	0.601	0.605	0.618
Inter-group consistency adjusted distance	0.028	0.012	0.012	0.021	0.032
Intra-group consistency-adjusted distance	0.027	0.020	0.022	0.022	0.030
Overall PRI	0.576				
Overall Consistency	0.785				

Although Original Coverage and PRI fluctuate in the robustness tests, they stay within acceptable bounds: coverage varies between 0.203 and 0.254, meaning the five pathways together still account for approximately 60 per cent of high-revenue cases (Overall Coverage = 0.576) without substantial erosion due to parameter shifts. PRI values range from 0.411 to 0.618; Path n2’s PRI (0.411) is marginally below 0.5 but, in most specifications, exceeds 0.60, suggesting that most pathways effectively avoid “pseudo-sufficiency.” Notably, Paths n3 and n5 consistently maintain PRI above 0.60, further confirming the robust explanatory power of the “subsidy + low-cost + high-green” core configuration.

Regarding the inter-group/intra-group consistency-adjusted distance, all pathways display values below 0.035—well under the 0.10 alert threshold—indicating that inter-path differences are not distorted by threshold tightening or loosening and that each configuration’s causal links remain stable both within and between groups. In sum, whether considering consistency, coverage, PRI or adjusted-distance metrics, the five identified pathways offer highly robust explanations for high Proportion of Revenue from Older Adults Care Investment. On this basis, the study confidently employs these configurations for subsequent mechanism exploration and policy recommendations, providing a dependable empirical paradigm for green financing and investment decisions in the international older adult care sector under diverse regional conditions.

### 3.5 Regional coverage analysis

To further reveal the applicability and explanatory power of each configuration under different regional conditions, we calculated the regional coverage (Country Coverage) of each typical pathway (Configurations 1–5) based on samples from China’s Eastern, Central and Western regions. Table 5 presents the coverage values, which indicate the proportion of high-revenue cases in each region explained by a given configuration—a higher value denoting stronger representativeness.

**Table 5:** Coverage analysis.

Country coverage	Configuration 1	Configuration 2	Configuration 3	Configuration 4	Configuration 4
Eastern China	0.318	0.411	0.273	0.368	0.412
Central China	0.235	0.377	0.325	0.327	0.346
Western China	0.216	0.136	0.218	0.319	0.331

In the Eastern region, Configuration 5 (0.412) and Configuration 2 (0.411) exhibit the highest coverages, followed by Configuration 4 (0.368), Configuration 1 (0.318) and Configuration 3 (0.273). This suggests that, in the economically advanced and institutionally mature Eastern region, the “high-incentive + high-regulation + high-ageing + high-GDP + high-cost + high-green” combination (Configuration 5) and the “high-regulation + high-GDP + high-investment – low-green” pattern (Configuration 2) best explain providers’ high revenue shares. Eastern local governments possess strong green-incentive and regulatory capacity, and residents’ willingness and ability to pay rank among the nation’s highest, facilitating the widespread replication of these models. Conversely, Configuration 3 (“subsidy + low-regulation – low-cost – high-green”) has the lowest coverage in the East, indicating its weaker applicability in this region.

In the Central region, Configuration 2 (0.377) and Configuration 5 (0.346) also show high coverage, followed by Configuration 4 (0.327) and Configuration 3 (0.325), while Configuration 1 (0.235) is markedly lower. Compared to the East, the Central region’s reliance on the high-regulation + economy-driven pathway (Configuration 2) is almost identical, reflecting strong momentum in infrastructure investment and market-based regulation. Notably, Configuration 3’s coverage in the Central region rises to 0.325—significantly above the East’s 0.273—indicating that fiscal subsidies and low-cost financing likewise effectively stimulate green investment and revenue growth in Central provinces. Thus, the Central region can both adopt the East’s scale-and-regulation model and leverage fiscal incentives for green transformation.

The Western region displays a more dispersed pattern of pathway applicability. Configuration 5 (0.331) and Configuration 4 (0.319) maintain relatively high coverage, whereas Configuration 3 (0.218) and Configuration 1 (0.216) are lower, and Configuration 2 (0.136) is minimal. This suggests that, given the West’s relatively underdeveloped economy and uneven policy implementation, the “economic + scale” model (Configuration 4) and the “holistic policy–market–firm synergy” model (Configuration 5) better fit local realities. With limited market capacity and government support, providers can still achieve high revenues through large-scale infrastructure or comprehensive policy and green investment synergies. In contrast, the “high-regulation + high-GDP + high-investment – low-green” pathway (Configuration 2) is almost inoperative in the West, underscoring the need to strengthen environmental regulation and high-end market demand locally.

The Eastern, Central and Western regions thus exhibit clear differences in configuration coverage: the East relies more on high-GDP and high-regulation models; the Central region benefits from both regulation-driven and subsidy-driven pathways; the West must balance scale expansion with policy–market–firm synergy. Such regional heterogeneity suggests that policymakers and enterprise managers should tailor strategies to local conditions: the East may continue to enhance regulation and market-based allocation; the Central region can pursue dual incentives of subsidies and regulation; and the West should integrate scale expansion with green incentives to achieve sustainable development and improved returns in the older adult care industry.

## 4 Discussion

This study, adopting an international perspective and employing dynamic QCA, systematically reveals the multivariate configuration pathways through which seven policy–market–firm variables influence the Proportion of Revenue from Older Adults Care Investment. First, necessity analysis confirmed that no single variable constitutes a necessary condition for high (or low) revenue share, validating the “multiple causes, multiple effects” and “equifinal” complexity of older adult care performance. Configurational pathway analysis then identified five typical modes—namely “high-subsidy + high-cost – high-green,” “high-regulation + high-GDP – low-green,” “subsidy + low-cost – high-green,” “scale-intensive capital” and “comprehensive synergy.” Each exhibited robust overall consistency (0.801) and coverage (0.576), and robustness tests confirmed that consistency remained above 0.80 under various parameterisations, with only minor shifts in coverage.

Regional coverage analysis further uncovered the geographic heterogeneity of pathway applicability: the Eastern region is typified by high-regulation and high-GDP driving modes; the Central region can both adopt the East’s hard-infrastructure and regulation model and benefit from “subsidy + green” combinations; the Western region relies more on scale expansion and comprehensive policy–market–firm synergy. These differences imply that, across development stages and institutional contexts, older adult care incentive tools and investment strategies must be locally adapted to maximise policy and market effects (11).

## 5 Conclusion and implications

### 5.1 Conclusion

This study, framed around “sustainable financing for ageing-related industries,” adopts an international perspective and specifies a seven-variable core system—local government tax relief incentives for green building initiatives (A), Regional Environmental-Regulation Stringency (B), Degree of Population Ageing (C), Regional GDP per Capita (D), Interest Rate on Loans to Firms in the Older Adults Care Sector (E), Gross Fixed-Asset Investment by Firms (F), and Proportion of Environmental Expenditure by Firms (G)—which is systematically mapped onto the Structure–Conduct–Performance (SCP) framework. Necessity analysis indicates that no single factor constitutes a necessary condition for either high or low revenue share, corroborating the domain’s conjunctural and equifinal causal structure: performance outcomes are not determined by unidirectional variation in any single indicator but instead emerge from particular combinations of structural variables (D, C) and conduct variables (A, B, E). Accordingly, both theoretically and empirically the evaluation of policy instruments should proceed from a configurational perspective—assessing tool effectiveness and priority conditional on structure—while policy practice should favour context-sensitive bundles of measures rather than uniform prescriptions derived from average-effect regressions.

To identify these configurations and their temporal dynamics, the study applies Dynamic fuzzy-set QCA (Dynamic fsQCA) to an annual, province-level panel of the seven variables, performing fuzzy calibration and truth-table analysis to extract typical high-revenue configurations. Five robust high-revenue paths are uncovered: (i) a “high-subsidy + high-ageing + high-cost + high-green” policy-driven green-transition pattern; (ii) a “high-regulation + high-economic development + high-investment – low-green” regulation-and-scale driven pattern; (iii) a “high-subsidy + low-regulation – low-cost – high-green” incentive-activation pattern; (iv) a “high-economic development + high-cost + large-scale – low-green” scale-expansion pattern; and (v) an extreme “policy–regulation–market–economy fully-coordinated” synergy pattern. These configurations perform robustly on key QCA metrics—consistency ranges from 0.811 to 0.845, raw coverage from 0.213 to 0.270, and PRI from 0.412 to 0.623—and remain stable after sensitivity checks on calibration thresholds, minimum case frequency, and repeated sub-sample analyses: the aggregate consistency stays above 0.80 and coverage shows only minor fluctuation. Methodologically, Dynamic QCA here not only delineates the sufficient/necessary structure of condition combinations but, via sequence comparison, reveals configuration switches—i.e., the temporal evolution of paths as regulation, market conditions, and demographic structure change—thereby addressing limitations of conventional regression approaches in capturing conjunctural causation and temporal path dependence.

Regional coverage analysis further reveals pronounced spatial heterogeneity in path applicability across eastern, central, and western regions. The eastern region is dominated by the “full coordination” and “high-regulation + high-economic development” patterns, indicating that in economically advanced and institutionally mature contexts, regulation and long-term financing are the principal levers for magnifying green investment effects. The central region exhibits greater adaptability, able both to adopt regulation-driven paths typical of developed areas and to achieve green premiums via targeted subsidies. The western region relies more on scale expansion and balanced policy–market–firm coordination, with policy impacts frequently contingent on synchronized fiscal support and market cultivation. Based on these findings, we offer context-sensitive policy recommendations: in high-D settings prioritize strengthening regulatory frameworks and long-term financing mechanisms to reduce E and secure returns on green investment; in C-driven markets emphasise demand stimulation and lower entry barriers to unlock market potential; and in mixed contexts deploy interactive policy packages with careful attention to sequencing. The study contributes methodologically and theoretically by (1) integrating Dynamic QCA with the SCP framework to provide a toolset for identifying multiple causal paths and their dynamic evolution, (2) empirically validating and refining the field’s conditional-dependence and equifinality characteristics, and (3) delivering replicable financing–investment portfolio templates of practical relevance for local governments and older adults care providers. Limitations include Dynamic QCA’s requirement for adequate temporal density (which constrains sequence interpretation with sparse short panels), the interpretive demands of complex Boolean solutions (which benefit from qualitative triangulation), and data availability constraints that limit finer-grained or cross-national generalisation. Future research could combine firm-level panel data, case interviews, and high-frequency temporal QCA, or apply synthetic control/causal-inference methods, to further validate the universality of identified paths and to assess long-run policy effects.

### 5.2 Theoretical implications

Previous research on older adult care and green building has tended to focus on a single domain—environmental economics, public policy or geroeconomics—lacking systematic integration (9). This study is the first to incorporate regional factors (green incentives, environmental regulation, ageing) and firm-level variables (financing cost, fixed-asset investment, environmental investment share) into a unified framework, thereby mapping the multi-dimensional policy–market–firm interaction mechanism. It also pioneers the use of dynamic QCA in international older adult care research, enabling the simultaneous identification of multiple sufficient and necessary condition combinations and capturing their temporal evolution—an innovative methodological paradigm for complex socioeconomic inquiries.

Traditional regressions and structural-equation models seek a single optimal solution or average effect, making it difficult to reveal diverse causal mechanisms across countries and stages (49). By validating five typical configurations, this study confirms the applicability of the “equifinality” theory in older adult care green financing and demonstrates that multiple paths—such as “high-subsidy + high-green,” “high-regulation + high-scale” and “capital-intensive expansion”—can all lead to high revenue share. Necessity and robustness analyses further substantiate the absence of any single necessary condition in complex systems, providing a solid empirical case for applying complexity theory in public administration and industry studies.

Regional coverage analysis shows that configuration effectiveness varies significantly with economic development and policy context: the East relies on comprehensive synergy models; the Central region benefits from both regulation-driven and subsidy-driven paths; the West emphasises scale expansion and policy–market synergy. This finding echoes “policy mix” theory, suggesting that green transformation in older adult care requires more than a single incentive or regulation—regions must flexibly configure tax incentives, regulatory stringency and financing instruments, coordinated with firm behavior, to form a complementary supply–demand–market loop. This theoretical extension offers innovative perspectives for policy design and regional development strategy.

### 5.3 Limitations and future research

Despite its innovations in older adult care financing and green investment research, this study has several limitations. Variations in data definitions for green incentives, regulatory intensity and corporate environmental expenditure across countries mean calibration cannot entirely eliminate cross-region measurement error, which may influence dynamic QCA outcomes. We measure firms’ financing cost solely by average older adult care industry loan rates, without distinguishing green loans from conventional ones, limiting analysis of green financing instruments’ diversity and premium effects. Due to inconsistencies in public databases and corporate reporting, some variables—such as regulatory intensity—rely on secondary sources, whose precision requires validation through fieldwork or expert interviews (55).

Future research could introduce more granular financial indicators—green bond issuance, sustainable-loan quotas and corporate credit ratings—to examine how specialised green instruments affect investment decisions and revenue performance. Incorporating non-financial performance dimensions—service quality, customer satisfaction and social impact—would enable an “economic–social–environmental” triple-bottom-line evaluation, using mixed methods (quantitative + qualitative) to assess each configuration’s contribution to overall sustainability (31).

Moreover, future studies should undertake cross-cultural and in-depth case comparisons by selecting representative countries or cities for field surveys and interviews, analysing how institutional culture, stakeholder interactions and local practices shape configuration formation and boundary conditions. Through richer empirical evidence and complementary multi-level methods, the theoretical framework for green financing and risk management in the older adult care industry can be further refined, providing more targeted and actionable policy and practice recommendations for sustainable development under global ageing (56, 57).

## Data Availability

Publicly available datasets were analyzed in this study. This data can be found at: https://www.stats.gov.cn/.
